# Effects of repetitive transcranial magnetic stimulation of the right inferior parietal lobe on the body image perception in anorexia nervosa: A pilot randomized controlled study

**DOI:** 10.1002/brb3.3617

**Published:** 2024-07-05

**Authors:** Nathalie Chastan, Najate Achamrah, Olivier Etard, Clément Nathou, Marie‐Astrid Piquet, Sébastien Guillaume, Jérôme Attal, André Gillibert, Pierre Dechelotte, Olivier Guillin, Marie‐Laure Welter

**Affiliations:** ^1^ Department of Neurophysiology Normandie University, UNIROUEN, Rouen University Hospital Rouen France; ^2^ Department of Nutrition Normandie University, UNIROUEN, INSERM UMR1073, Rouen University Hospital Rouen France; ^3^ Department of Neurophysiology Normandie University, UNICAEN, INSERM, COMETE, CYCERON, CHU Caen Caen France; ^4^ Department of Psychiatry CHU Caen Caen France; ^5^ Department of Nutrition CHU Caen Caen France; ^6^ Department of Emergency Psychiatry and Acute Care Lapeyronie Hospital, CHU Montpellier, Institute of Functional Genomics, University of Montpellier, CNRS, INSERM Montpellier France; ^7^ Department of Biostatistics CHU Rouen Rouen France; ^8^ Department of Psychiatry Normandie University, UNIROUEN, Rouen University Hospital Rouen France

**Keywords:** anorexia nervosa, body image, repetitive transcranial magnetic stimulation, right inferior parietal lobe

## Abstract

**Introduction:**

Restrictive anorexia nervosa (AN) is associated with distorted perception of body shape, previously linked to hypoactivity and reduced excitability of the right inferior parietal lobe (rIPL). Here, we investigated the impact of high‐frequency repetitive transcranial magnetic stimulation (HF rTMS) of the rIPL on body shape perception in patients with AN.

**Methods:**

Seventeen patients with AN (median [Q1_Q3] age, 35 [27_39] years; disease duration, 12 [6_18] years) were randomly assigned to receive real or sham HF (10 Hz) rTMS of the rIPL over a period of 2 weeks, comprising 10 sessions. The primary outcome measure was the Body Shape Questionnaire (BSQ). Secondary outcomes included eating disorder symptoms, body mass index, mood, anxiety, and safety. Data collection were done at baseline, post‐rTMS, and at 2 weeks and 3 months post‐rTMS.

**Results:**

Following both real and sham rTMS of the rIPL, no significant differences were observed in body shape perception or other parameters. Both real and sham rTMS interventions were deemed safe and well tolerated. Notably, serious adverse events were associated with the underlying eating and mood disorders, resulting in hospitalization for undernutrition (five patients) or suicidal attempts (two patients).

**Conclusion:**

This pilot study does not support the use of rTMS of the rIPL as an effective method for improving body shape perception in individuals with the restrictive form of AN. Further research is warranted to comprehensively explore both the clinical and neurophysiological effects of HF rTMS in this population.

## Graphical abstract

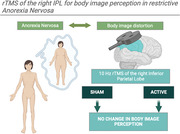

## 1 INTRODUCTION

Anorexia nervosa (AN), a prevalent eating disorder among adolescents, manifests through severe food restriction leading to undernutrition and is associated with significant morbidity, including mood disorders, and an almost sixfold increase in mortality rates (Arcelus et al., 2011; van Eeden et al., 2021). In addition to food restriction, AN involves an intense fear of gaining weight or becoming fat, persistent behaviors that interfere with weight gain, and a distorted body image or lack of recognition of the seriousness of low body weight (Dobrescu et al., 2020). This body image distortion significantly contributes to the perpetuation of food restriction and refusal to acknowledge thinness (Dobrescu et al., 2020). Current treatments, such as pharmacological and cognitive behavioral therapies, have shown limited effectiveness, resulting in a chronic course of the disorder in about 20% of cases (Dobrescu et al., 2020).

The intricate pathophysiology of AN remains poorly understood. Recent research has identified various structural and functional brain anomalies, including decreased gray matter volume in the anterior and median cingulate cortex, left middle occipital gyrus, and inferior parietal lobe (IPL) (Joos et al., 2010; Su et al., 2021; Titova et al., 2013). Additionally, disruptions in resting state functional connectivity in the anterior and median cingulate cortex (Su et al., 2021), as well as white matter alterations within the thalamocortical and occipital‐parietal‐temporal‐frontal tracts, have been reported (Gaudio et al., 2019). Regarding body image distortion, abnormal activation has been observed in the lateral temporo‐occipital, precuneus, and inferior parietal cortices (Gaudio et al., 2019), with a positive correlation of “drive for thinness” and gray matter volume of the right IPL (Joos et al., 2010). Hypoactivation of the rIPL during tasks involving the visualization of one's body relative to others (Vocks et al., 2010), coupled with increased amygdala activity during visualization of others’ bodies or one's own body being inflated (Vocks et al., 2010; Wagner et al., 2003), was also reported in AN patients and linked to heightened emotional sensitivity and social comparison awareness (Gaudio & Quattrocchi, 2012).

In line with these recent advances, non‐invasive brain stimulation has emerged as a potential therapeutic option for treating AN. Repetitive transcranial magnetic stimulation (rTMS) has shown potential in modulating cortical excitability, with low‐frequency (<5 Hz) rTMS decreasing and high‐frequency (≥5 Hz) rTMS increasing cortical excitability (Fitzgerald et al., 2006). High‐frequency rTMS targeted at the dorsolateral prefrontal cortex (DLPFC) has shown promise in alleviating certain clinical symptoms, such as reducing subjective feelings of being “fat” or “full” (Van den Eynde et al., 2013) and fat avoidance (Muratore et al., 2021). However, controlled studies performed on larger cohorts have produced mixed results (Dalton et al., 2018; McClelland, Bozhilova, Campbell, et al., 2013; McClelland, Bozhilova, Nestler, et al., 2013), indicating varying effects on mood and anxiety (Dalton et al., 2018). A recent open‐label study found that 20–30 sessions of high‐frequency rTMS targeted at the dorsomedial prefrontal cortex (PFC) in AN patients with comorbid depression improved functional scales of eating disorders and comorbid anxiety (Woodside et al., 2021). A meta‐analysis suggested only moderate therapeutic effects on depressive and anxiety symptoms, with no impact on body mass index (BMI) (Cavicchioli et al., 2022). This highlights the heterogeneous nature of rTMS protocols, including targeted brain areas, session durations, inter‐train intervals, and the number and frequency of train pulses (Cavicchioli et al., 2022).

In this study, considering the reduced rIPL activity observed in AN patients and its link to body image distortion, we hypothesized that enhancing the excitability of the rIPL through high‐frequency rTMS could potentially ameliorate body image distortion in individuals with AN.

## 2 PATIENTS AND METHODS

### 2.1 Study design and patients

In this randomized double‐blind parallel group trial, patients were assigned to receive either real or sham 10 Hz rTMS across 10 sessions. The recruitment of participants was carried out at the Psychiatry and Nutrition departments at the Rouen, Caen, and Montpellier University Hospitals. Inclusion criteria were as follows: right‐handed adults (age ≥ 18 years old), diagnosed with AN of the restricting subtype, as per the Diagnostic and Statistical Mental Disorders (Call et al., 2013). Additionally, individuals were required to have a disease duration of over 1 year, a BMI of ≤18.5 kg/m^2^ sustained for a minimum of 3 months, and a stable medical drug regimen maintained for >3 months. To be eligible for the study, patients willingly consented and provided informed written consent, and were affiliated with a social security scheme. Non‐inclusion criteria were as follows: contraindications to rTMS or Magnetic resonance imaging (MRI) (pace‐maker, neurostimulation, metallic implants anywhere in the body, etc.), pregnancy, lactation, a history of seizures or an increased risk thereof, prior exposure to rTMS, or the presence of any brain lesion. This study was done in accordance with the Declaration of Helsinki and good clinical practice guidelines. It was approved by the local ethics committee and was registered on ClinicalTrials.gov (NCT01717079).

### 2.2 Randomization and masking

Patients were randomly assigned to either real or sham rTMS group, with a ratio of 1:1 between the two trial arms. Randomization was overseen and conducted by the Rouen University Clinical Research Unit, without stratification. Both the patients and the investigators remained blinded to the treatment allocation throughout the study. The neurologist responsible for administering the rTMS sessions was not involved in patient assessments.

### 2.3 Procedures

Patients underwent a comprehensive assessment at the inclusion before TMS (baseline, D0), followed by the first rTMS session (Figure). Over the course of a 2‐week period, a total of 10 rTMS sessions were administered. The 10th rTMS session was followed by a second assessment (Figure). Subsequently, two follow‐up visits were scheduled at 2 weeks and 3 months post‐treatment (Figure).

Before the rTMS sessions, every patient underwent a structural MRI scan to precisely localize the right inferior parietal lobe (IPL, Broadmann area 40, supramarginalis gyrus) for neuronavigation purposes (eXimia NBS, Nexstim). Each rTMS session commenced by determining the patients’ motor threshold (MT) through the recording of the left thumb adductor muscle using surface Electromyography (EMG) electrodes and using a real TMS figure‐of‐eight coil applied over the right primary motor cortex. The motor threshold was defined as the minimum stimulator output intensity required to obtain a peak‐to‐peak amplitude of the motor‐evoked potentials >50 μV for half of a series of 10–20 stimulations.

The Magstim Rapid device and the Magstim 70‐mm air‐cooled real and sham coils were employed to perform rTMS sessions. Patients allocated to the real group received 10 sessions of 10 Hz frequency at 90% of their individual MT. In line with previous published trials (Dalton et al., 2018; McClelland, Bozhilova, Campbell, et al., 2013), each session consisted of 20 trains of 10 s with 50‐s inter‐train intervals, resulting in a total of 2000 pulses per session, all precisely targeted at the right IPL. In the sham group, a similar stimulation protocol was applied, using a sham coil designed to replicate the sensory experience of the active coil without delivering actual stimulation to the brain, ensuring the integrity of the masking procedure.

### 2.4 Outcomes

The primary outcome measure was the Body Shape Questionnaire‐34 (BSQ‐34). This is a one‐dimensional questionnaire consisting of 34 items to assess concerns about body weight and shape. Responses are rated on a six‐point Likert scale (“never,” “rarely,” “sometimes,” “often,” “very often,” and “always”), with the total scores ranging from 34 (no body image concerns) to 204 (severe concerns) (Cooper et al., 1987; Rousseau et al., 2005).

Several secondary outcomes were assessed, including the Eating Attitude test (EAT‐40), a 40‐item self‐report questionnaire measuring dieting behavior, oral control, food preoccupation, and body intake (Garner & Garfinkel, 1979); the Bulimia test (BULIT), a 36‐item self‐report questionnaire assessing signs of bulimia (Thelen et al., 1996); the Eating Disorder Inventory (EDI‐2), a 91‐item self‐report questionnaire evaluating the presence of eating disorders through 11 subscales: “drive for thinness,” “bulimia,” “body dissatisfaction,” “ineffectiveness,” “perfectionism,” “interpersonal distrust, interoceptive awareness,” “maturity fears,” “asceticism,” “impulse regulation,” and “social insecurity” (Garner, D. M., 1991); and the Hamilton Anxiety and Depression Rating Scales (HARS and HDRS) to assess symptoms of anxiety and depression (Hamilton, 1959, 1967).

Physical indicators such as the Body Mass Index (BMI = body mass, kg/[height, cm]^2^) and percentage of fat mass and lean mass measured via Dual Energy X‐ray Absorptiometry (DEXA) using a Lunar Prodigy device (GE Medical Systems Lunar) were also included.

Safety and tolerability were also assessed after each rTMS session and at each visit. Any new symptom was considered an adverse event and classified as serious if it resulted in hospital admission, persisted as sequelae, or was deemed critical by the attending clinician.

### 2.5 Statistics

To evaluate the impact of the intervention, we utilized non‐parametric Mann–Whitney tests to compare scores for the BSQ, EAT‐40, BULIT, EDI‐2, HARS, HDRS, SF‐36, and BMI measurements between the patients’ groups at both baseline and after the rTMS. The analysis was conducted in an intention‐to‐treat (ITT) manner, with all randomized patients included in the primary analysis and data imputed using the “last observation carried forward” (LOCF) method for missing data pertaining to the primary outcome. For imputation, median values of both randomization groups at baseline were employed. Additionally, a post hoc analysis was conducted, considering differences at baseline, utilizing a linear model to explain immediate post‐stimulation BSQ (primary endpoint) by the baseline BSQ and by the ITT randomization group, with LOCF, mirroring the approach in the main analysis. All statistical analyses were conducted with the R statistical software (the R Foundation for Statistical Computing), with a significance set at.05.

## 3 RESULTS

### 3.1 Sample description

A screening of 20 patients with AN resulted in the enrollment and randomization of 17 participants, with a median age of 35 years (range: 27–39), a disease duration of 12 years (range: 6–18), and a BMI of 16 kg/m^2^ (range: 14–17) (Table 1). Three patients withdrew from the study before randomization: two of these patients withdrew their approval, and one was hospitalized for a medical issue before randomization, resulting in premature dropout. Among the randomized patients, seven were allocated to the real rTMS group and 10 to the sham rTMS group. Baseline analysis showed significant differences between the groups, with higher scores for the EDI‐2 and BULIT scales, as well as BMI, in patients assigned to the sham rTMS group compared to those in the real rTMS group (Table 1).

**TABLE 1:** Baseline characteristics and effects of real and sham repetitive transcranial magnetic stimulation (rTMS) of the right inferior parietal lobe (IPL) in 17 patients with Anorexia nervosa.

	Baseline		*p*‐value	Post‐rTMS		*p*‐value
	Real	Sham		Real	Sham	
Age (years)	36 (29_39)	29 (27_36)				
Disease duration (years)	18 (7_21)	11 (7_12)				
BSQ‐34	93 (67_142)	154 (130_164)	0.055	85 (54_90)	120 (95_151)	0.073
EAT‐40	42 (34_55)	72 (61_78)	0.082	44 (33_50)	65 (48_70)	0.28
EDI‐2	81 (47_88)	130 (110_141)	0.016	69 (40_85)	108 (76_115)	0.057
BULIT	68 (55_76)	100 (92_114)	0.003	67 (59_73)	90 (83_114)	0.001
HARS	16 (8_23)	16 (14_30)	0.46	10 (6_16)	12 (6_16)	0.96
HDRS	16 (14_18)	16 (13_20)	0.96	12 (11_13)	12 (10_14)	1.0
BMI	13.6 (13.2_15.5)	16.5 (15.6_17.1)	0.025	14 (13_16)	16 (15_18)	0.073

*Note*: Values are median (Q1_Q3); *p*‐value: between‐group comparison at baseline and post‐rTMS sessions.

Abbreviations: BMI, body mass index; BSQ‐34, body‐shape questionnaire‐34; BULIT, Bulimia test; EAT‐40, eating attitude test; EDI‐2, eating disorder inventory; HARS, Hamilton Anxiety Rating Scales; HDRS, Hamilton Depression Rating Scales.

### 3.2 Effects of real or sham IPL rTMS on AN symptoms

**FIGURE 1:**
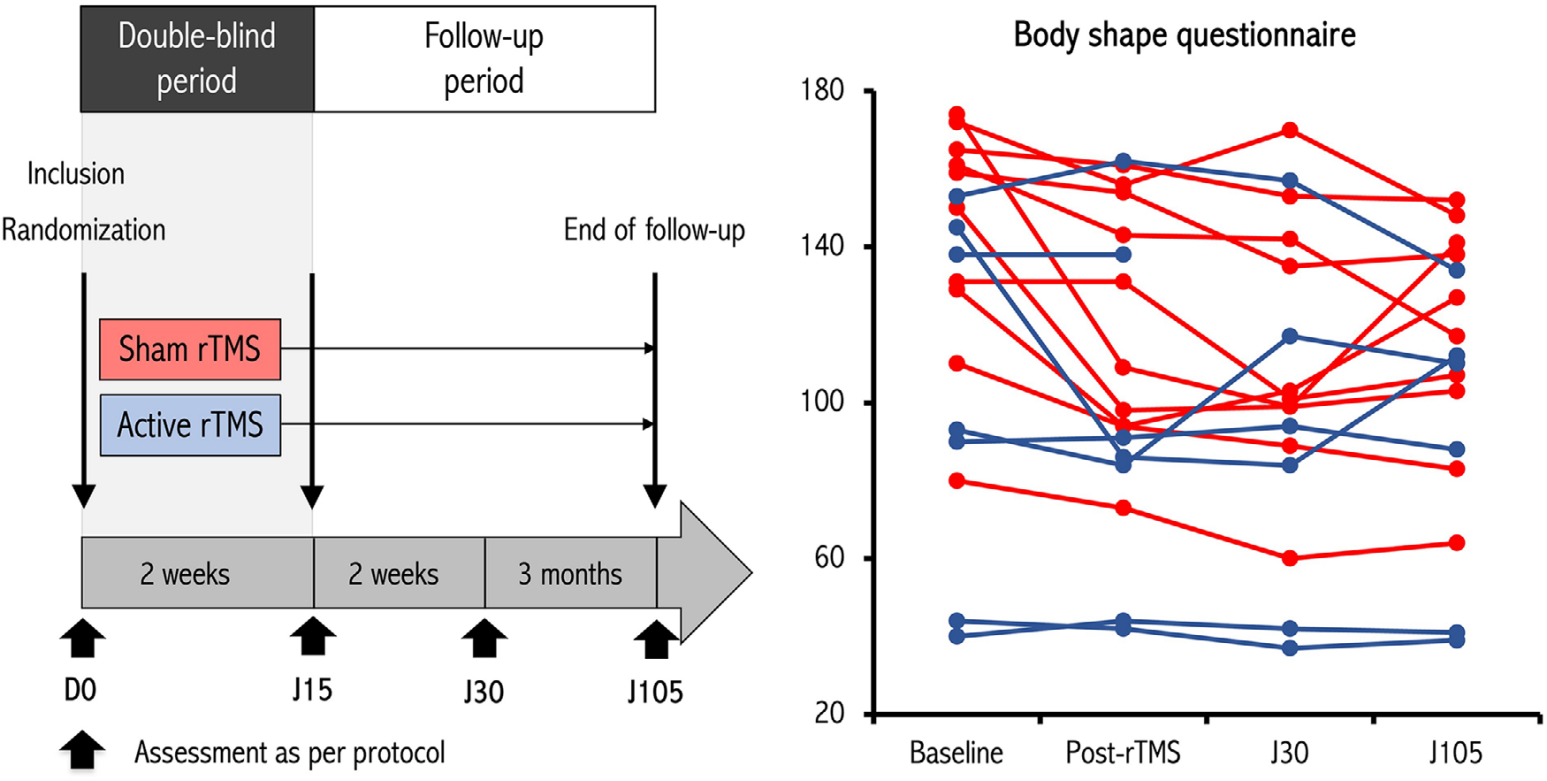
Study design and effects of real and sham high‐frequency repetitive transcranial magnetic stimulation (rTMS) of the right inferior parietal lobe (IPL) on the Body Shape Questionnaire in patients with Anorexia Nervosa (AN). Study design (left panel) and Body Shape Questionnaire scores obtained in AN patients randomized in the real (blue) or sham (red) groups. Each line represents one individual patient.

Comparing the scores between the real and sham rTMS groups post‐rTMS, we found no significant differences in BSQ‐34 (Figure 1), EAT‐40, EDI‐2, HARS and HDRS scores, or BMI values (Table 1). The BULIT score remained significantly higher in the sham group compared to the real rTMS group (Table 1).

Within‐group analysis revealed no significant changes in the BSQ‐34, ETA40, EDI2, HARS and HDRS scores, or BMI values, after either sham or real rTMS (Table 1). The changes in the BSQ‐34 score from baseline to post‐rTMS were also not significantly different between the two groups, with a mean effect of real versus sham rTMS of +5.6 points (linear model, 95% coefficient interval: −21.3 to +32.6, *p* = .66).

### 3.3 Follow‐up period

During the follow‐up period, assessments at 2 weeks and 3 months post‐rTMS sessions were conducted for 16 patients. No significant changes were observed in BSQ‐34 or other scores, or in the proportion of lean and fat masses in both groups (not shown). The BULIT score continued to exhibit significant differences between groups, with persistently higher scores in patients assigned to the real rTMS group (Table 1).

### 3.4 Adverse events

Throughout the rTMS sessions, a total of 30 non‐serious adverse events were reported, with 20 occurring in the sham group and 10 in the real group. Cephalalgia and twitching of the eyelid were reported during sham rTMS, while no rTMS related side effects were reported during real IPL rTMS sessions. During the randomized period, eight serious adverse events were documented, involving three patients assigned to the sham rTMS group and four in the real rTMS group. These events included five cases of exacerbated undernutrition and deteriorated general status, leading to hospitalizations in the psychiatry department for two patients, and enteral nutrition for one patient. Additionally, two patients committed a suicidal attempt through drug intoxication, requiring intensive care hospitalization and subsequent withdrawal from the study in one patient. Another patient developed a pulmonary infection, which resolved with appropriate antibiotic therapy.

## 4 DISCUSSION

This pilot‐controlled study is the first to examine the effects of high‐frequency rTMS targeting the right IPL on body image distortion in AN patients. Our results indicated that HF‐rTMS of the rIPL failed to statistically significant improvements in body image distortion or other related AN symptoms. Severe adverse events were observed across patient groups, primarily due to AN severity, with a good tolerance of the rTMS protocol.

The lack of observable effects on body shape distortion, but also BMI, anxiety, or depression, in our AN patients could be attributed to several factors. First, the patients randomized to the real rTMS group had very severe disease profiles, with significant body image distortion, extremely low BMI (mean <14), long disease duration, and resistance to previous interventions. In prior therapeutic trials assessing the effects of rTMS in AN patients, the average BMI was around 16 (Dalton et al., 2018; Muratore et al., 2021; Woodside et al., 2021), whereas it was 13.6 in our patients randomized to real rTMS. This severe baseline condition might have limited the potential benefits of our approach. Another factor could be the chosen protocol. Although previous studies that tested HF rTMS of the DMPFC used similar protocols, with a 10 Hz frequency and a total number of pulses of 20,000 delivered over a 2‐ or 3‐week period (Dalton et al., 2018; McClelland, Bozhilova, Campbell, et al., 2013, McClelland, Bozhilova, Nestler, et al., 2013; Woodside et al., 2021), this might not have been optimally designed to modulate the targeted brain area's excitability. A longer treatment period might be necessary for substantial changes (Dalton et al., 2018), rather than focusing solely on the total number of pulses administered. Unlike previous rTMS studies targeting the DLPFC, we observed no significant changes in anxiety or mood. In fact, mood worsened in two patients who attempted suicide after rTMS, one in each patients group, with no clear relationship to the intervention. However, our protocol targeting the rIPL did not aim to improve these psychiatric comorbidities. Overall, these data indicate that targeting the rIPL alone may not suffice for significant changes in body perception, despite its involvement in self‐other discrimination and self‐perception processes (Karhson et al., 2015; Uddin et al., 2006). Due to the complex fronto‐parietal neural network disruptions involved in AN, prior research suggests that targeting the prefrontal cortical areas might be more effective in modulating these functional networks. This approach has shown a more favorable impact on clinical and behavioral components (BMI, food avoidance) (Dalton et al., 2018), and IPL activity modulation (Ćurčić‐Blake et al., 2022). When applied over the DMPFC, rTMS induced significant improvements in mood, anxiety, and some core domains of AN pathology, including shape and weight concerns. This improvement was correlated with lower baseline functional connectivity between the DMPFC, right inferior pole, and left angular gyrus, parts of the “salience network” (Woodside et al., 2021). This was interpreted as a lesser capacity for cognitive control over perseverance or negative self‐referential thinking in responsive AN patients. When applied over the DLPFC, rTMS also induced significant improvements in anxiety and mood, but no changes in body image distortion or BMI. Overall, these data could suggest that improvements in mood, anxiety, and eating behaviors may precede improvements in body image distortion.

This study also acknowledges several limitations. First, the small cohort size limits the generalizability of the findings. Second, the two groups exhibited significant differences in AN severity at baseline, which could have affected the statistical comparison of the effects of rTMS between groups. Additionally, the absence of an assessment of the neurophysiological effects of the HF‐rTMS protocol hinders a comprehensive understanding of its impact on local and network brain excitability and functional connectivity.

## 5 CONCLUSIONS

This small cohort study does not advocate for the use of HF‐rTMS of the right IPL to ameliorate body image distortion in patients with severe AN. Further studies are necessary to optimize protocols, considering less severe cases and identifying the most effective target areas for intervention in AN. Future research incorporating neurophysiological markers will provide insights into the relationship between brain activity changes and symptom improvement, advancing more effective therapeutic approaches for AN patients.

## AUTHOR CONTRIBUTIONS

**Nathalie Chastan**: Conceptualization; funding acquisition; investigation; methodology; project administration; resources; supervision; visualization; writing—original draft; writing—review and editing. **Najate Achamrah**: Conceptualization; investigation; project administration; supervision; writing—review and editing. **Olivier Etard**: Investigation; project administration; writing—review and editing. **Clément Nathou**: Investigation; project administration; writing—review and editing. **Marie‐Astrid Piquet**: Investigation; project administration; writing—review and editing. **Sébastien Guillaume**: Investigation; project administration; writing—review and editing. **Jérôme Attal**: Investigation; project administration; writing—review and editing. **André Gillibert**: Data curation; formal analysis; funding acquisition; methodology; validation; visualization; writing—original draft; writing—review and editing. **Pierre Dechelotte**: Investigation; project administration; writing—review and editing. **Olivier Guillin**: Conceptualization; funding acquisition; investigation; project administration; supervision; writing—review and editing. **Marie‐Laure Welter**: Investigation; methodology; project administration; supervision; visualization; writing—original draft; writing—review and editing.

## CONFLICT OF INTEREST STATEMENT

The authors declare no conflicts of interest.

### PEER REVIEW

The peer review history for this article is available at https://publons.com/publon/10.1002/brb3.3617.

## Data Availability

All relevant data are within the article. Requests for anonymized data should be sent to Marie‐Laure Welter at the CHU Rouen, 76000 Rouen, France.
